# Comorbidity of asthma and hypertension may be mediated by shared genetic dysregulation and drug side effects

**DOI:** 10.1038/s41598-019-52762-w

**Published:** 2019-11-08

**Authors:** Olga Zolotareva, Olga V. Saik, Cassandra Königs, Elena Yu. Bragina, Irina A. Goncharova, Maxim B. Freidin, Victor E. Dosenko, Vladimir A. Ivanisenko, Ralf Hofestädt

**Affiliations:** 1 Bielefeld University, International Research Training Group “Computational Methods for the Analysis of the Diversity and Dynamics of Genomes” and Genome Informatics, Faculty of Technology and Center for Biotechnology, Bielefeld, Germany; 2 Institute of Cytology and Genetics, Siberian Branch of Russian Academy of Sciences, Novosibirsk, Russia; 3 Bielefeld University, Bioinformatics and Medical Informatics Department, Bielefeld, Germany; 4 Research Institute of Medical Genetics, Tomsk NRMC, Tomsk, Russia; 5 Bogomoletz Institute of Physiology, Kyiv, Ukraine

**Keywords:** Data integration, Medical genomics

## Abstract

Asthma and hypertension are complex diseases coinciding more frequently than expected by chance. Unraveling the mechanisms of comorbidity of asthma and hypertension is necessary for choosing the most appropriate treatment plan for patients with this comorbidity. Since both diseases have a strong genetic component in this article we aimed to find and study genes simultaneously associated with asthma and hypertension. We identified 330 shared genes and found that they form six modules on the interaction network. A strong overlap between genes associated with asthma and hypertension was found on the level of eQTL regulated genes and between targets of drugs relevant for asthma and hypertension. This suggests that the phenomenon of comorbidity of asthma and hypertension may be explained by altered genetic regulation or result from drug side effects. In this work we also demonstrate that not only drug indications but also contraindications provide an important source of molecular evidence helpful to uncover disease mechanisms. These findings give a clue to the possible mechanisms of comorbidity and highlight the direction for future research.

## Introduction

Asthma and hypertension affect hundreds of millions of people worldwide^1,2^ and coincide in adults more frequently than expected by chance. Patients with asthma are more likely to have high blood pressure^3^ and, in turn, the presence of hypertension is associated with the increased severity of asthma^4^. This association between asthma and hypertension was confirmed in multiple studies of different patient cohorts^3–7^ but its underlying causes remain unknown. The coexistence of two or more diseases, called comorbidity, was shown for many human disorders^8–10^. Moreover, not only complex disorders demonstrate comorbidity but also several Mendelian diseases coincide with complex disorders^10,11^. Comorbidity can be direct or inverse^8,12^, when the presence of one disease protects from the development of another one. For example, the coincidence of neoplasms with several nervous system disorders is lower than expected^13,14^.

Comorbidity may point to causal relationships between two diseases. For instance, hypertension may cause cardiovascular damage, leading to consequences such as heart failure, stroke, and kidney problems^2^. Alternatively, comorbidity may result from confounder effects, e.g. lifestyle or environmental factors, predisposing to multiple health problems. For instance, smoking is a risk factor for multiple diseases^15^, including hypertension^2^ and asthma^1^. Also, stress may trigger asthma attack^1^, and long-term stress exposure is associated with the risk of hypertension^2^. Finally, comorbidity of two diseases may be an effect of a third disease. For instance, obesity is another common risk factor for asthma and hypertension and decreasing of the body-mass index is helpful for management of both diseases^16,17^.

In addition to these risk factors, some anti-asthmatic drugs may worsen hypertension and vice versa, and several anti-hypertensive drugs are contraindicated in asthma. For example, beta-blockers used to control blood pressure can cause asthma attacks^18,19^ and therefore are contraindicated for asthma patients. At the same time, beta-agonists, used for treatment of asthma may increase heart rate^20^ and should be used with caution in patients with hypertension. Corticosteroids are aimed at suppressing the immune system and are used against asthma^1^, but can at the same time elevate blood pressure due to their effect on kidneys, leading to enhanced liquid retention^21^.

Nevertheless, comorbidity between asthma and hypertension can only in part be explained by excessive weight, smoking and the use of specific drugs and persists after consideration of these variables, although becomes weaker^4^. Besides environmental risk factors, including other diseases and drugs, comorbidity may arise as the result of shared molecular genetic basis^22^, predisposing the patient to the development of both diseases. Comorbid diseases may share associated genes themselves^23–25^ or demonstrate strong connectivity between two sets of associated genes in protein interaction^11,26–29^, gene coexpression^26^ or metabolic networks^30^.

Recent studies have shown that many epidemiologically correlated diseases and traits share risk loci identified in genome-wide association studies (GWAS)^31,32^, but this is not true for hypertension and asthma^31^. However, GWAS results are not the only information source of gene-disease associations. Many different approaches are used to establish associations between genes and diseases. For example, genes altering expression in disease and genes encoding drug targets are likely to be also involved in its pathogenesis.

Despite the fact that many genes are associated with isolated asthma and hypertension, molecular mechanisms underlying their comorbidity remain unclear. To investigate the genetic basis of this comorbidity, we constructed comprehensive lists of asthma and hypertension associations of different nature and identified genes implicated in both diseases. We analyzed the relationships between these genes, revealed six tightly interconnected gene modules and characterized these modules by enriched GO terms, pathways and tissue specificity. Identification of the genes and gene modules potentially involved in both disorders may advance the unraveling the mechanisms of their comorbidity, and help get further insight into the pathogenesis of asthma and hypertension comorbidity.

## Results

### Genes previously associated with asthma and hypertension

Although the phenomenon of comorbidity of asthma and hypertension has long been known, only a few works discussing genes potentially involved in both diseases or related pathophysiological processes have been published before 2018. We have found six publications which discussed ten genes in total potentially relevant to both asthma and hypertension and summarized the results of our literature search in Table 1. These studies were mostly focused on genes and their functions and did not aim to discover the mechanisms of comorbidity between the two diseases.

**Table 1:** Genes potentially involved in the pathophysiology of asthma and hypertension, according to literature published before 2018.

Gene	Functions	Evidence
*TLR4*	pathogen recognition and activation of innate immunity	Up-regulated in lungs of spontaneously hypertensive rats (SHR) compared to normotensive ancestor strain (WKY rats) in response to combustion source particulate matter treatment which irritates lungs^96^
*CXCL2* (*MIP-2*)	suppress hematopoietic progenitor cell proliferation	
*CD14*	mediates the innate immune response to bacterial lipopolysaccharides	
*RHOA*	reorganization of the actin cytoskeleton and regulation of cell shape, attachment, and motility	Up-regulated in rodent models of asthma and hypertension; inhibition leads to improvement of both conditions^97^. ROCK inhibitors suppress smooth muscle contraction and may treat arterial hypertension and asthma^98^
*ROCK1*	regulates formation of focal adhesions	
*GNA12, GNA13*	signal transduction	These genes encode G-protein subunits transducing the signal from activated GPCR to RhoGEFs activating *RHOA*. Abnormal G12/13 signaling is involved in the pathogenesis of arterial hypertension and bronchial asthma, among other pathophysiological conditions^99^
*SLC26A4*	encodes transmembrane anion exchanger	Madeo *et al*.^100^ reported a variant of *SLC26A4* with potentially protective effect for both asthma and hypertension
*ADRB1*, *ADRB2*	mediate the physiological effects of the epinephrine and norepinephrine	These genes encode proteins targeted by drugs used against asthma and hypertension; some variants are associated with response to anti-hypertensive^101^ anti-asthmatic therapy^102^

In our recent work^33^, we applied ANDSystem^34,35^ to perform the automatic reconstruction of the associative gene network for asthma and hypertension from scientific literature. The resultant associative network included 205 genes potentially responsible for comorbid asthma and hypertension. We proposed gene prioritization methodology based on ten criteria including relevance scores calculated by state-of-the-art gene prioritization tools^36,37^, association with biological processes, position in the associative network and evidence of genetic associations and regulations. The *IL10*, *TLR4*, and *CAT* genes had the highest ranks among all candidate genes, and *ADRB2* was ranked sixth. Our later works demonstrated experimental evidence supporting possible roles of *IL10* and *TLR4* in comorbid asthma and hypertension. Drevytska *et al*. created an animal model of comorbid asthma and hypertension – SHR with OVA-induced asthma – and demonstrated that *IL10* knock-down in this model improves cardiac and lung function parameters^38^. Bragina *et al*. identified expression quantitative trait loci (eQTL) associated with asthma and hypertension comorbidity for *CAT*, *TLR4*, *ANG* and *RNASE4* genes on the cohort of 587 individuals from West Siberia^39^. More recently, Saik *et al*. reanalyzed associative asthma/hypertension network and, based on the cross-talk centrality, proposed ten more immune-related genes for further experimental validation^40^.

All the previous works investigated the genetic basis of asthma/hypertension comorbidity evaluated the relevance of single genes. In contrast with previous studies, in this work, among all the associations extracted from a variety of data sources, we identified and characterized groups of functionally related genes. Our approach aligns with current understanding of asthma and hypertension as complex disorders, which thought to be polygenic and heterogeneous^41,42^. Yet another advantage of this work is that we incorporated types of associations not considered in previous studies nor provided by large gene-disease association portals, such as Open Targets^43^ or DisGeNET^44^. In particular, we included into the analysis genes controlled by eQTLs associated with asthma and hypertension and target genes of drugs contraindicated in these diseases.

### The overlap between genes associated with asthma and hypertension independently

Aiming to compose comprehensive lists of genes associated with asthma and hypertension and investigate their overlap, we collected gene-disease associations supported by a variety of evidence types: genetic associations, differential expression, co-occurrence in texts, and targeting by drugs used against asthma and hypertension. Also, to expand these gene lists, we used GWAS hits mapped to known eQTLs and selected genes regulated by these eQTLs. Finally, we hypothesized, that genes targeted by drugs causing adverse effects similar to asthma and hypertension may also be involved in their pathogenesis. Therefore, in addition to drugs that can treat asthma and hypertension, we took into account drugs that exaggerate these diseases and added their targets in the lists of genes for asthma or hypertension.

**Figure 1:**
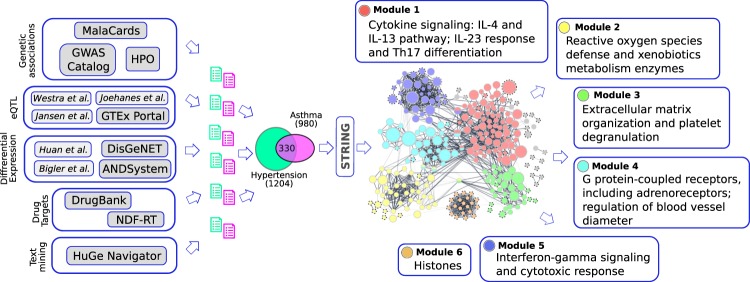
Identification and characterization of gene modules associated with asthma and hypertension. Network nodes represent genes and are colored according to membership in a module. Nodes not assigned to clusters are shown in grey. Size of each node is proportional to the number of evidence sources supporting the association of corresponding gene with asthma or hypertension. All gene set overlap analysis results are shown in Supplementary Tables S2A–C.

After combination of gene-disease associations from various evidence sources (see Methods and Fig. 1), the lists of 980 genes associated with asthma and 1204 genes associated with hypertension were compiled. Out of them, 330 genes were associated with both diseases according to at least one data source (Supplementary Table S1). To facilitate further use of associations relevant for asthma and hypertension comorbidity, we added them to GenCoNet database^45^.

### Network analysis of shared genes reveals six modules

In order to identify groups of functionally related genes, we built a functional network of shared asthma/hypertension genes using Cytoscape^46^ v3.6.1 stringApp^47^ v1.3.0 and performed module detection. In this network, two nodes were connected, if corresponding genes are co-expressed or if proteins physically interact or share biological pathways. Only edges representing gene co-expression, physical interactions of encoded proteins and biological pathway sharing, were included in the network. Overall, 257 out of 330 genes (78%) were connected with at least one of other genes. Six tightly interconnected modules were detected using ClusterViz App^48^ v1.0.3 EAGLE algorithm^49^ (with minimal Clique Size and Complex Size thresholds set to 10) and annotated with overrepresented pathways, GO terms, and tissue-specific gene sets (Supplementary Tables S2A–C provide all significant gene set overlap analysis results).

Module 1 comprised 77 genes was enriched with genes participating in cytokine signaling, in particular, in *IL-4*, *IL-13*, and *IL-23*, and Th17 differentiation pathways. *IL-4* and *IL-13* regulate inflammatory response to allergen exposure in asthma^50^. *IL-23* and Th17 cells are elevated in hypertension^51^ and promote neutrophilic inflammation in asthma^52^. A very recent review on the treatment of comorbid asthma and hypertension summarizes implications of cytokine signaling and Th17 cells for both diseases^53^. Module 2 comprised 43 genes participating in the metabolism of xenobiotics and defending the cell from oxidative stress. Reactive oxygen species are generated by airway epithelium cells exposed to irritants, activate immune cells and thought to be implicated in asthma^54^. Hypertension is characterized by elevated oxidative stress biomarkers in blood and increased oxidative damage of vasculature^55,56^. A large body of evidence confirms roles of antioxidant defence enzymes in asthma^57–59^ and hypertension^60,61^. Interestingly, a key antioxidant enzyme, catalase (*CAT*), was also previously associated with comorbid asthma and hypertension^33,39^. Module 3 enriched with genes involved in extracellular matrix organization and platelet degranulation. Platelets are altered in hypertension^62^ and their release products participate in airway inflammation and remodeling in asthma^63^. Module 4 was enriched by genes encoding G protein-coupled receptors and responsible for regulation of blood vessel diameter. This module included adrenoreceptor genes, many of which are targeted by drugs against asthma and hypertension. Module 5 was enriched with genes participating in cytokine signaling. In contrast to module 1, genes from module 5 were associated with interferon gamma response and more specific for CD16-monocytes and sputum, than for neutrophils. Interferon-gamma inhibits Th2-induced inflammation but promotes cytotoxic response^64^. Interferon gamma signaling is implicated in asthma^64^ and angiotensin-II-induced hypertension^65^. Module 6 was composed of genes encoding histones, whose modifications define chromatin state and regulate gene expression. Changes of histone modifications are shown in many diseases including asthma^66^ and hypertension^67^. Inhibition of histone deacetylases decreases inflammations^68^ and demonstrate anti-hypertensive effects^67,69^.

Some modules overlapped in a few genes: *MAPK1* and *EDN1* belonged to modules 1 and 4, *C3* and *C5* to modules 2 and 4, *STAT1* to modules 1 and 5, and *SERPINA1* to modules 2 and 3. Such genes may be responsible for a cross-talk between biological processes represented by modules these genes connect. However, of these six genes, only *MAPK1* and *STAT1* were associated with asthma or hypertension through evidence other than co-occurrence in texts.

### Analysis of evidence types supporting gene-disease associations

**Figure 2:**
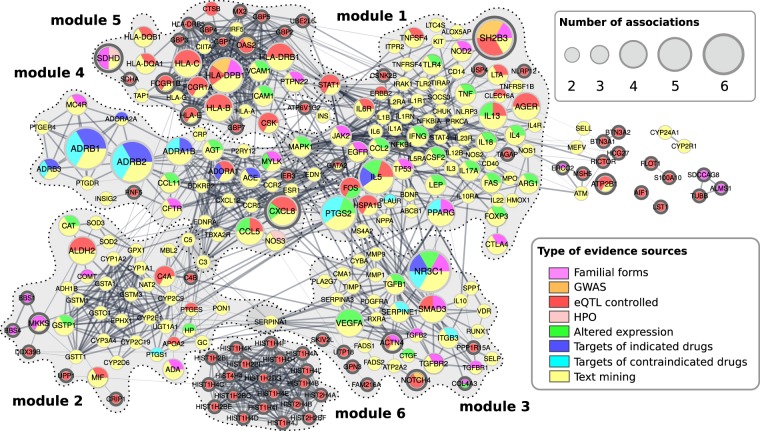
Evidence sources supporting gene associations with asthma and hypertension. In this figure, we used node style similar to Fig. 1B in^103^. Here, nodes represent genes associated with both asthma and hypertension, edges correspond gene interactions (only 257 nodes connected with at least one other node are shown). Genes are colored according to evidence sources (see figure legend) from which associations came from. The size of each node is proportional to the number of evidence sources supporting its association with asthma and hypertension. All 330 genes associated with asthma and hypertension annotated with evidence types supporting associations are listed in Supplementary Table S1.

To investigate the contribution of various evidence types to the detection of shared genes, we labeled every gene with the kind of relationship, linking it with asthma or hypertension, and analyzed the distribution of labels among six modules. Figure 2 shows that the distribution of labels over the network does not seem to be random, and the representations of different evidence type labels among modules are not equal. The majority of shared genes (231 of 330) had an association established via text mining by HuGE Phenopedia^70^ v2.1 and were supported by at least two independent studies. Moreover, associations of 125 of them were established via text mining only and constituted essential parts of modules 1–4. Although frequent co-occurrence in texts does not seem to be the most confident evidence of association, we could not exclude it from consideration without losing 206 (62%) of shared genes.

We also found that all members of module 6 and almost all of module 5 included genes that are controlled by GWAS-identified eQTLs associated with asthma or hypertension. In total, 107 out of 330 shared genes were regulated by such eQTLs. Since this number significantly exceeded the expected by chance (Fisher’s exact test p-value < 6.94e-145; permutation p-value < 10e-4; 10 expected on average), we can hypothesize that these shared genes may have an impact on the development of comorbidity between asthma and hypertension. Importantly, this observation cannot be explained simply by shared GWAS hits. Only one missense variant, rs3184504 in *SH2B3* gene, was in the intersection of asthma and hypertension GWAS associations. It was independently associated with hypertension, blood eosinophil count and many other traits and controlled only 39 of 107 genes associated with asthma and hypertension through eQTL regulation. Interestingly, among these 39 genes 5 were up-regulated in blood of patients with hypertension and included in the “blood pressure signature” proposed by Huan *et al*.^71^: *FOS*, *MYADM*, *PPP1R15A*, *S100A10*, and *TAGAP*.

To illustrate the novelty of our findings, we compared our set of shared genes with gene sets obtained from Open Targets v18.02 and DisGeNET v5.0 using trivial disease names. In our list of 330 shared genes, 82 were novel (nodes shown in bold frames in Fig. 2), i.e. not associated with asthma and hypertension in OpenTargets and DisGeNET. These two large gene-disease association databases use distinct sets of evidence sources and interpret some associations differently. For example, they do not consider gene regulation data for establishing gene-disease associations, although it is implicated in asthma^72^ and hypertension^71^. Open Targets and DisGeNET include gene-disease associations obtained directly from GWAS Catalog, where disease-associated variants are mapped to neighboring genes and do not differentiate synonymous and non-synonymous variants. As a result, most of the newly identified shared genes were associated via eQTLs and composed modules 5 and 6.

### Adverse drug reactions may mediate comorbidity of asthma and hypertension

We observed more common drugs (and therefore drug targets) relevant to asthma and hypertension than expected by chance. We composed four lists of drugs that influence asthma or hypertension (Supplementary Table S3): drugs with positive effects on asthma or hypertension (i.e. used to treat or relieve their symptoms) and drugs with negative effects on these diseases (i.e. contraindicated or worsening disease symptoms). Eight drugs used to treat hypertension were harmful for asthma patients: timolol, nadolol, sotalol, pindolol, carvedilol, labetalol, propranolol. All these drugs belong to the class of non-selective beta-blockers and may exacerbate asthma^19^. At the same time, seven anti-asthmatic drugs occurred in the list of drugs that may increase the risk of hypertension or elevate blood pressure: triamcinolone, prednisolone, methylprednisolone, dexamethasone, hydrocortisone (corticosteroids) and epinephrine, ephedra, ephedrine (beta-agonists). Both overlaps were statistically significant with p-value < 2.64e-06 and p-value < 4.50e-06, respectively in Fisher’s exact test.

**Figure 3:**
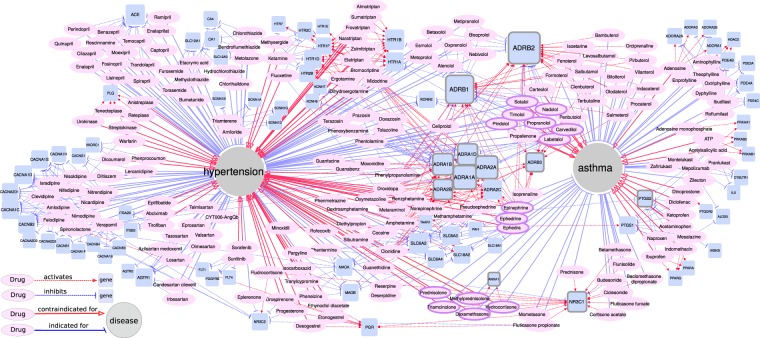
Relationships between genes and drugs indicated and contraindicated in asthma and hypertension. All target genes significantly overrepresented in one of four drug groups are shown. Drugs influencing both diseases and target genes overrepresented in more than one group are shown with bold frames.

To find target genes whose activation or inhibition exhibit positive or negative influence on asthma and hypertension, we performed target overrepresentation analysis in four drug groups. 96 genes were significantly overrepresented among targets of at least one of four drug groups (Table 2 and Supplementary Table S4), only 16 of which were in asthma-hypertension network. Interestingly, 8 of these 16 genes associated with asthma or hypertension through drug evidence appeared in module 4, enriched with genes involved in smooth muscle contraction. Figure 3 summarizes all significant drug targets, their relationships with drugs, and drug effects on asthma and hypertension. Supplementary Table S5 provides this network in tabular format. *ADRB1* and *ADRB2* were targeted by drugs from all the four groups, and activation and inhibition of beta-adrenoreceptors caused opposite effects on asthma and hypertension. Similarly, *NR3C1*, a glucocorticoid receptor, was activated by drugs indicated in asthma but potentially harmful for hypertension. *ANXA1* is another target of corticosteroid drugs, such as dexamethasone and hydrocortisone, its activation mediates the anti-inflammatory effect via inhibition of phospholipase A2^73^. *ANXA1* is up-regulated in blood of hypertensive patients^71^. *PTGS2* inhibition seemed to have a negative effect on both diseases, while *PTGS1* was overrepresented only among targets of drugs contraindicated in asthma.

**Table 2:** Genes overrepresented among targets of drugs influencing asthma and hypertension.

Disease	Drug Group	# Drugs	# Targets	Drug Targets Overrepresented in the Group
asthma	indications	61	81	*ADORA1*, *ADORA2A*, *ADORA2B*, *ADORA3*, ***ADRB1***, ***ADRB2***, ***ADRB3***, ***ANXA1***, *CYSLTR1*, *HDAC2*, *IL5*, ***NR3C1***, *PDE3A*, *PDE4A*, *PDE4B*, *PDE4C*
asthma	contraindications	45	142	*ABCC1*, *ABCC2*, ***ADRB1***, ***ADRB2***, *AKR1C3*, *ALOX5*, *IKBKB*, *KCNQ2*, *KCNQ3*, *PPARA*, *PPARG*, *PRKAA1*, *PRKAB1*, *PRKAB2*, *PTGDR2*, *PTGS1*, ***PTGS2***
hypertension	indications	99	128	*ACE*, ***ADRA1A***, ***ADRA1B***, ***ADRA1D***, ***ADRA2A***, ***ADRA2B***, ***ADRB1***, ***ADRB2***, *AGTR1*, *AGTR2*, *BDKRB1*, *CA1*, *CA4, CACNA1B*, *CACNA1C*, *CACNA1D*, *CACNA1F*, *CACNA1H*, *CACNA1S*, *CACNA2D1*, *CACNA2D2*, *CACNA2D3*, *CACNB1*, *CACNB2*, *CACNB3*, *CACNB4*, *CACNG1, KCNH2*, *KCNH6*, *KCNH7*, *NR3C2*, *SCNN1A*, *SCNN1B*, *SCNN1D*, *SCNN1G*, *SLC12A1*, *SLC12A3*
hypertension	contraindications	94	188	***ADRA1A***, ***ADRA1B***, ***ADRA1D***, ***ADRA2A***, ***ADRA2B***, *ADRA2C*, ***ADRB1***, ***ADRB2***, ***ADRB3***, ***ANXA1***, *FGA*, *FLT1*, *FLT4*, *HTR1A*, *HTR1B*, *HTR1D*, *HTR1E*, *HTR1F*, *HTR2B*, *HTR2C*, *HTR7*, *ITGA2B*, *ITGB3*, *MAOA*, *MAOB*, ***NR3C1****, PAH*, *PDGFRB*, *PGR*, *PLAUR*, *PLG*, ***PTGS2***, *SERPINB2*, *SERPINE1*, *SLC18A1*, *SLC18A2*, *SLC6A2*, *SLC6A3*, *SLC6A4*, *TAAR1*, *VKORC1*

Target genes significantly enriched in more than one group are shown in bold. Genes *ADRB1* and *ADRB2* whose potential role in asthma and hypertension was previously discussed are underlined.

Our results agree with prior knowledge about asthma and hypertension medications. It is well known that $\beta$-blockers and agonists have opposite effects on asthma and hypertension. Although medications for treating hypertension and asthma target different receptors (*ADRB1* blockers have anti-hypertensive effect^2^ and *ADRB2* agonists have anti-asthmatic effect^1^), there are many components non-specifically targeting multiple types of adrenoreceptors^19^. Blockers of ${\alpha }_{1}$ and agonists of ${\alpha }_{2}$-adrenergic receptors cure hypertension^2^, and opposite actions on these receptors may provoke blood pressure elevation. Alternative treatment options for asthma patients who poorly respond to beta-agonists are inhaled or systemic glucocorticoids^74^. Overdosage of glucocorticoids induces elevation of blood pressure, although inhaled glucocorticoids are relatively safe compared to systemic steroids because of their local action^21^. Patients with an elevated level of glucocorticoids due to their overproduction or abnormal metabolism develop Cushing syndrome^75^, characterized by high blood pressure among other symptoms. Other classes of anti-inflammatory drugs, such as non-steroid anti-inflammatory drugs (NSAID) may also influence asthma and hypertension. NSAID-Exacerbated Respiratory Disease (NERD), is a classic example of adverse drug reaction in response to aspirin and some other NSAIDs. NERD is also called aspirin-induced asthma because it resembles main asthma symptoms, such as bronchial obstruction and dyspnea, and is prevalent among asthma patients^76^. NERD is thought to result from *PTGS1* inhibition, therefore the usage of *PTGS2* selective inhibitors is considered to be relatively safe for asthmatics^77^, but it is associated with a risk of hypertension^78,79^. Also, NSAIDs may decrease the effect of anti-hypertensive medications^80^.

## Discussion

In this study, we investigated the genetic overlap between a pair of comorbid disorders, asthma and hypertension. A correlation of gene perturbation with disease status points to the possible involvement of this gene in the disease mechanism. At the same time, different molecular lesions may have similar effects on the phenotype. These considerations motivated us to construct two sets of genes independently associated with each disease through various perturbation types and investigate their overlap. We found 330 genes simultaneously associated with both diseases and potentially responsible for their comorbidity. Projecting these shared genes to an interaction network revealed six functional modules comprising tightly interconnected genes. We tested these modules for the overrepresentation of GO terms and pathways, tissue-specificity and evidence types supporting gene associations.

We observed an excess of genes jointly controlled by asthma- and hypertension-associated eQTLs in modules enriched with genes involved in interferon-gamma signaling and chromatin assembly. This observation suggests that the coincidence of asthma and hypertension may be at least partially explained by concordantly altered genetic regulation of certain biological processes of functions. Our finding agrees with very recent results published by Li *et al*.^81^, who analyzed multiple disease pairs and demonstrated that comorbid diseases share significantly more eQTL-regulated transcripts than expected by chance.

We also found more drugs with opposite effects on asthma and hypertension than expected by chance. Based on this observation, we hypothesized that comorbidity might be the result of drug side effects, when drugs against one disease may predispose the patient to the development of another. This particular case of asthma and hypertension demonstrates that genes targeted by contraindicated drugs may also participate in pathophysiologic mechanisms of comorbidity.

An important limitation of this study is the absence of direct validation of the resulting associated gene set. Since the genetic basis of asthma and hypertension comorbidity is poorly understood, we have no gold standard to compare with. Another way of result evaluation would be an experimental validation of gene roles in the comorbidity, which was not in the scope of this work.

The validity of our results is partially confirmed by the presence of some known shared genes in our set. Four genes (*ADRB1*, *ADRB2*, *TLR4*, and *CD14*) listed in Table 1 appear among 330 genes associated with asthma and hypertension. This limited overlap may be explained by the fact that most of the papers included in Table 1 do not focus on asthma or hypertension and only list them among multiple phenotypes associated with certain genes. Therefore, if associations of these genes with asthma and hypertension do not appear in abstracts, they remain invisible for text mining tools.

Another indirect confirmation of the result validity is our observation that comorbid diseases share many genes controlled by eQTLs. This agrees with the results of Li *et al*.^81^, obtained independently on different datasets.

## Methods

### Monogenic associations via hereditary asthma and hypertension

From MalaCards^82^ v4.5, and literature^2,83^ we extracted genes that carry mutations causing monogenic syndromes with asthma or hypertension among other symptoms. In total, we found 37 genes associated with familial forms of hypertension or with Mendelian disorders characterized by hypertension. No monogenic forms of asthma were found, although several Mendelian syndromes characterized by frequent asthma attacks considered in the literature^83^. From human phenotype ontology^84^ (HPO) we selected phenotype terms related to asthma (HP:0002099 - Asthma, HP:0012042 - Aspirin-induced asthma, HP:0025428 - Bronchospasm) and hypertension (HP:0000822 - Hypertension, HP:0000875 - Episodic hypertension, HP:0004421 - Elevated systolic blood pressure, HP:0004972 - Elevated mean arterial pressure, HP:0005117 - Elevated diastolic blood pressure). We excluded genes associated with specific kinds of hypertension (e.g. ocular hypertension) that are not related to essential hypertension (HP:0007906, HP:0001409, HP:0002092, HP:0002640, HP:0008071, HP:0100817, HP:0005168). Mapping of genes to HPO terms was downloaded from http://compbio.charite.de/jenkins/job/hpo.annotations.monthly/lastStableBuild/, table ALL_SOURCES_FREQUENT_diseases_to_genes_to_phenotypes.txt” available on 26.01.2018. We used gene-phenotype pairs marked as “frequent”, which means that this phenotype was manifested in at least 50% patients with a disease.

### Coding GWAS variants

GWAS Catalog^85^ v1.0.1 (downloaded on 22.08.2017) includes 29 studies related to asthma which report 407 associations in total (395 unique). In addition, we included 17 associations from 4 studies of asthma-related traits such as NERD, eosinophil count or Immunoglobulin E (IgE) levels. Similarly, 120 associations with hypertension and blood pressure traits were obtained from 16 studies listed in GWAS Catalog. All associations have p-value < 10e-5 (default in GWAS Catalog). Only 29 and 9 single-nucleotide polymorphisms (SNPs) associated with asthma and asthma-related traits and with hypertension and blood pressure respectively were annotated as splice_region_variant, missense_variant, synonymous_variant or non_coding_transcript_exon_variant and therefore affected transcript sequences.

### Regulation

Although only a small fraction of variants discovered in GWAS affects transcripts, about a half of them overlap with eQTLs^86^. From three recent blood eQTL studies^86–88^ comprising thousands of individuals, we obtained lists of eQTL SNPs and overlapped them with variants associated with asthma and hypertension in GWAS. Furthermore, we included variants associated with tissue-specific expression from GTEx v6^89^. This dataset contained eQTLs in 44 tissues including whole blood. The details of eQTL data sources used in this study are presented in Table 3. From each study, we retained only SNP-gene pairs which passed the false discovery rate (FDR) threshold of 0.05. All SNPs coordinates were lifted over to hg38 and their IDs were converted into dbSNP^90^ v150 to match SNPs IDs used in GWAS catalog.

**Table 3:** Characteristics of eQTL datasets.

Dataset	samples	Tissue or cell type	Cis-eQTLs	Trans-eQTLs
GTEx v6	449	44 tissues including whole blood	639825	24
Westra *et al*.^87^	5311	PBMC	397310	349
Joehanes *et al*.^86^	5257	whole blood	2072003	149592
Jansen *et al*.^88^	4896	whole blood	1212555	6913

### Differentially expressed genes

We obtained asthma and hypertension expression signatures from the two biggest expression profiling studies of blood pressure^71^ and asthma^91^. From Huan *et al*. we took 34 genes with expressions associated with hypertension diagnosis or with systolic and diastolic blood pressure. Asthma expression signature included 541 genes differentially expressed in the blood of asthma patients in Bigler *et al*. dataset. In addition to these two signatures, we composed two lists of genes that demonstrated altered expression levels in asthma or hypertension according to ANDSystem (published on 09.10.2014) and DisGeNET v5.0. These two resources provide collections of differentially expressed genes automatically extracted from biomedical literature. To reduce false positives, we took genes whose altered expression in asthma and hypertension was supported by at least two evidence sources.

### Drugs and drug targets

Drugs indicated and contraindicated in asthma and hypertension were obtained from DrugBank^92^ v5.0.9 and NDF-RT^93^ (released on 02.10.2017). Surprisingly, 5 anti-asthmatic drugs (budesonide, flunisolide, salmeterol, formoterol, and cromoglicic acid) were at the same time contraindicated in asthma according to NDF-RT. These drugs are known to cause paradoxical drug reactions, for example, when the intake of an anti-asthmatic drug provokes bronchospasm^94^. We excluded such drugs from contraindications list but kept them in the list of indications, because paradoxical reactions are extremely rare. Finally, the list of contraindicated drugs was extended with drugs reported to induce the rise of blood pressure and asthma, NERD or bronchospasms. From DrugBank, we obtained genes targeted by selected drugs. All target genes encoding non-human proteins were excluded. All drug action types used in DrugBank were converted to simplified effects reflecting the direction of drug action on its target, e.g. “positive” for agonists and activators, “negative” for inhibitors and antagonists, and “other” for modifiers. Supplementary Table S3 contains selected drugs, their effects on diseases and their targets.

### Statistical tests

Gene set overrepresentation tests were carried out in TargetMine^95^ (build 20180327) with default parameters (one-tailed Fisher’s exact test followed by Benjamini-Hochberg correction for multiple testing; p-value threshold 0.05; all genes from the tested database were considered as the background set). Similarly, overrepresentation of drug targets in groups of drugs evaluated using the one-sided Fisher’s exact test implemented in scipy python library. We considered all the drugs from DrugBank with defined action on any human target as the background, and applied p-value threshold of 0.05 after Benjamini-Hochberg correction for multiple testing.

To demonstrate that asthma and hypertension share more eQTL regulated genes than expected by chance, we generated 10000 pairs of random eQTL sets and calculated the number of shared genes for each pair. Sizes and overlap of randomly generated eQTL sets were set the same as real sets of asthma and hypertension eQTLs.

## Supplementary information

Dataset 1

Dataset 2A

Dataset 2B

Dataset 2C

Dataset 3

Dataset 4

Dataset 5

## Data Availability

All data generated during this study are included in Supplementary Information files.
